# Clinical practice workflow in Radiation Oncology should be highly standardized

**DOI:** 10.1002/acm2.12555

**Published:** 2019-03-12

**Authors:** Per Halvorsen, Nilendu Gupta, Yi Rong

**Affiliations:** ^1^ Radiation Oncology Lahey Health Burlington MA USA; ^2^ Radiation Oncology Ohio State Univ Columbus OH USA; ^3^ Radiation Oncology University of California Davis Cancer Center Sacramento CA USA

## 1 INTRODUCTION

Radiotherapy (RT) treatment is a complex process that requires the entire radiation oncology team working together to ensure a safe, expeditious, and effective treatment of patients in the clinic. Standardizing the RT workflow is considered essential to improve RT treatment quality and reduce miscommunication or human errors. Our leading professional societies in medical physics and radiation oncology, that is the American Association of Physicists in Medicine (AAPM) and the American Society for Radiation Oncology (ASTRO), have invested tremendous resources in publishing task group reports, guidelines, and recommendations for best practice on almost every aspect of RT to ensure consistency and standardization. However, when it comes to every day routine clinical operation, different people might have different understanding or a proper workflow; and furthermore, there may be unpredictable scenarios that can potentially hinder the well‐established routine or standard RT workflow. The question raised in this debate is, should preset workflows be highly standardized and strictly followed? Herein, we have invited two experts in the field who both have distinguished themselves with years of experience in making these hard decisions in the clinic. Mr. Per Halvorsen argues for the proposition that “Clinical practice workflow in Radiation Oncology should be highly standardized”, while Dr. Nilendu Gupta shared some of his different opinions and experience.

Mr. Halvorsen is the Chief Physicist in Radiation Oncology at Lahey Health in suburban Boston. He received his MS in Radiological Medical Physics from the University of Kentucky in 1990 and was certified by the American Board of Radiology in 1995. He has been a member of the AAPM for nearly 30 yr and has been an active volunteer in professional societies, chairing the AAPM Professional Council and serving on the Board of Directors. He has authored numerous peer‐reviewed manuscripts, most recently as the chair of the Medical Physics Practice Guideline for Stereotactic Radiosurgery and Stereotactic Body Radiotherapy (SBRT) and as a member of the ASTRO‐ASCO‐AUA Evidence‐Based Guideline for Hypofractionated Prostate treatment. He is a volunteer surveyor for the American College of Radiology (ACR), and served many years on its accreditation program oversight committee. He is Deputy Editor‐in‐Chief of the open‐access Journal of Applied Clinical Medical Physics (JACMP), and a Fellow of the ACR and AAPM.

Dr. Nilendu Gupta is an Associate Professor of Clinical Radiation Oncology, the Chief of Medical Physics and the Director of the Medical Physics Residency Program in Radiation Oncology at The Ohio State University (OSU), James Cancer Hospital. Dr. Gupta received his Ph.D. in Biomedical Engineering at The Ohio State University. He plays a leadership role in quality and patient safety within the department and within the University and the OSU Health System.

## 2 OPENING STATEMENT

### 2.A Per H. Halvorsen

Do you think clinical workflows in Radiation Oncology are essentially the same across the country? If so, think again. I've had the privilege of conducting practice accreditation surveys across the country and have surveyed more than 50 centers in the last 2 decades, both large centers in cosmopolitan districts and single‐physician rural clinics. The variability in clinical workflows is striking.

Such variability carries risk.^1^ Ultimately, our highest priority is the patient.^2^ Our profession has a strong record of producing guidance documents for equipment‐based quality control (AAPM Task Group reports, ACR Technical Standards). After several process failures that caused significant patient harm and resulted in national media attention, the Radiation Oncology community belatedly recognized the risk of nonstandardized clinical workflows in the Safety Is No Accident publication,^3^ endorsed by 12 professional societies. Some years later, the AAPM's long‐awaited Task Group 100 report proposed a paradigm shift in quality management, toward prospective risk analysis with a focus on process.^4^

Routine clinical radiation oncology physics is a clinical service — not a “physics project”. Standardization of routine clinical workflows reduces risk^5^ and enhances efficiency.^6^, ^7^ Standardization facilitates the sharing of data to develop best practices.^8^ By standardizing routine clinical workflows, we create a more robust and efficient operation, enabling medical physicists to focus more attention on developing new procedures and validating new technologies.

Consider a simple example: Designing a conventionally fractionated treatment plan for a lung cancer patient to minimize the risk of cardiac toxicity. The physician requests that the mean heart dose be kept below 20 Gy in keeping with recently published data on cardiac toxicity.^9^ So, what should be included in the “heart” structure? A recent review of charts at our own institution found volumes for the “heart‐pericardium” structure varying from 125 to 1088 cc. Some people have “a big heart”, but that hardly explains this large variation. Instead, it is likely due to variability in contouring conventions and a lack of appreciation for how those contours are used to optimize a treatment plan (we promptly clarified and standardized our contouring convention upon this discovery).

Consider another common process: Simulation and treatment planning for external beam prostate cancer treatment. In several of the clinics I have surveyed for accreditation, each physician had their own process. Some used a bladder filling protocol for simulation, whereas others did not. Some used variable slice thickness scanning whereas others did not. Some physicians delineated organs at risk themselves whereas others requested that the dosimetrist do so. As a result, there were multiple workflows in the same clinic for the same treatment site. I can find no peer‐reviewed publication to show that such process variability and inefficiency results in better care for the patient.

Amid the ever‐increasing complexity of radiation oncology workflows, standardization provides clarity that improves our ability to spot potential problems and allows us to focus more of our energy on how to best leverage the power of modern technology for our patients’ benefit. We should embrace it.

### 2.B Nilendu Gupta, PhD

Over the past decade there has been heightened awareness of safety in Radiation Oncology,^10^ with a focus on radiation therapy workflow and processes, especially in identifying processes where break down due to appropriate checks and balances not being in place or not completed due to time pressures could result in potential compromize of safety of delivery of high doses of radiation to our patients.^11^ Safety systems and processes have been intensely studied and best practices from other disciplines applied to develop a better understanding and to develop appropriate interventions to enhance patient safety. Such efforts have resulted in mapping out various radiotherapy processes using systems engineering approaches as a first step toward developing better awareness within the multidisciplinary radiotherapy team. Other recent efforts have evolved around educating the radiotherapy community on developing and implementing risk based models to identify process steps that are crucial to patient safety or process breakdown to help focus our quality and safety efforts, for example the failure modes and risk analysis methodology presented in the AAPM TG‐100 report.^4^ Several radiotherapy practices have taken efforts a step further to implement best practices from the airline industry that boasts of one of the best safety records by implementing a safety culture involving checklists, timeouts and hard‐stops using tools such as Crew Resource Management (CRM).^12^

Being part of a large academic radiation oncology practice we have also implemented many of the tools outlined above over the past 6 years. These efforts included developing our own in‐house electronic whiteboards to initially collect process data and subsequently manage various radiotherapy processes, employing CRM principles to assign times to different steps and using our whiteboard software to track, remind and set alarm conditions when timelines are not met, along with identifying certain crucial hard stop conditions that are implemented specifically with our treatment planning workflows.^13^ As we have enforced such timelines and hard stops over the past several years, I feel that I cannot completely agree with the proposition as stated. I specifically disagree with the second half of the proposition about the workflows needing to be “highly standardized”. While process steps can be standardized very well, when it comes to process times it is much more difficult and arguably not possible to highly standardize them. I offer some specific comments and thoughts regarding my disagreement.

The efforts our field has made in researching and adopting best practices to enhance patient safety has been extremely beneficial. It has allowed different institutions to track and collect process data and allowed institutions to develop expectations of mean process times and times associated with sub steps and in turn provide guidance to the departmental management teams for developing standard operating procedures (SOPs). Such efforts have drastically changed operations of Radiation Oncology departments by helping us manage and invest in resources judiciously, while enhancing the safety of our operations. One inherent challenge with such process data collected is that the standard deviations for times associated with process steps are very high, stemming from a few factors. These factors can be broadly categorized into buckets such as variations of clinical circumstances from patient to patient, the decisions of how to separate out different complexities of processes and steps (e.g., 2D, 3D Conformal, IMRT, SBRT, etc. for treatment planning processes), and last but by no means least, human factors of team members within each department.^14^ To use data that have very high standard deviations to set process step times and highly standardize an operation is unfortunately not realistic and leads to innumerable operational challenges and are not easily surmountable. Assigning process times based on mean times essentially leads to a large percent of process steps that would inherently exceed the allowed time, and allowing added time for each step based on high standard deviation associated with the data would make the operation very inefficient and untenable.

In summary the efforts in our field toward quality and safety systems in radiotherapy processes have been very successful. Radiation oncology operations have become much more data driven and if implemented properly a clinic can have dashboards that provide operations data to help manage our operations more efficiently. Systems engineering approaches and tools from airline and other industries have allowed us to map our processes and process steps very well, identify the high risk steps to focus our efforts as well as develop checklists, timeouts and hard stops very successfully to enhance the safety of Radiotherapy operations and the treatments we deliver. However, to try to highly standardize processes, specifically process times based on data collected for a multiday processes in a complex operation is not very feasible. I feel that our efforts will be better invested in developing operations managers who can help manage our processes and direct resources real time to keep our operations most efficiently running, and also be a gatekeeper of making sure appropriate safety steps and hard stops are implemented. Such measures along with appropriate incentives/disincentives for managing human factors and patient satisfaction to departmental and health system expectations is the best way to manage our workflows.

## 3 REBUTTAL

### 3.A Per H. Halvorsen

Dr. Gupta provides a good summary of the recent focus on process and safety in our profession, and relates an admirable effort within his own clinical program in this regard. He outlines why standardized *time intervals* for key clinical processes are not practical. I agree with Dr. Gupta that it is not realistic to expect a tight distribution of process times in our practice environment. We're not manufacturing widgets, after all, we are optimizing individual patients’ treatments – and our patients’ medical histories are complex.

While it may not be feasible to achieve highly standardized process *times*, my focus is on the standardization of the procedure itself — ensuring that we have clear procedural instructions and clear and consistent documentation of the work product. In the absence of such structure, the result is inefficiency and higher potential for misinterpretation. To expand on my earlier examples: When a physician references published guidelines or national protocols as the basis for the treatment planning directives (which is good practice), the treatment planning should be performed consistent with those references, otherwise one risks misapplication leading to suboptimal treatment or worse. And when there is no consistent process for simulation of common treatment sites such as prostate, workflow is disrupted as staff members repeatedly ask physicians for per‐patient process instructions and patient confidence in the team is undermined as he observes the chaos. Of course, we must preserve the flexibility to tailor treatments to each patient's condition — but that can be accomplished with clear physician directives and consistent methods of chart documentation, following SOPs that clarify *how* key steps should be performed.

### 3.B Nilendu Gupta, PhD

I commend the passionate case my esteemed colleague has made in his opening statement, and believe we have a lot of areas of agreement in our views. We both agree on the fact that standardization of radiotherapy processes is essential to enhancing safety in our field. Per has made the case that process and practice standardization and uniformity is essential across every practice within our country. While I have not had the privilege of performing as many ACR site reviews my colleague has performed, I have also performed several over the past decade. While I do agree that the variability of processes are quite high from facility to facility and standardization would be of great benefit, some of the core recommendations to enforce standardization requires human, equipment, and financial resources that many facilities do not have the luxury of having at this time. I feel the first step toward accomplishing this goal is to follow the broader framework of national recommendations, but for each institution/facility to establish standardized processes within the resource constraints they have to work with. Over the next decade, as knowledge‐based contouring and shared knowledge‐based planning models and other tools become commonly available and used in all clinics, I am confident as a radiotherapy community we will be able to “highly” standardize our radiotherapy processes across the country. Meanwhile, we will have to continue to take baby steps toward getting to this goal.

## CONFLICT OF INTEREST

Authors have no conflict of interest to declare.
